# Local sympathetic nerve depletion does not alter vitiligo progression in a mouse model

**DOI:** 10.3389/fmed.2025.1466996

**Published:** 2025-01-31

**Authors:** Zhichao Hu, Ting Chen, Daoming Chen

**Affiliations:** ^1^Graduate School of Peking Union Medical College, Chinese Academy of Medical Sciences, Beijing, China; ^2^National Institute of Biological Sciences, Beijing, China; ^3^Tsinghua Institute of Multidisciplinary Biomedical Research, Tsinghua University, Beijing, China

**Keywords:** autoimmune disease, skin disease, vitiligo, sympathetic nerve, melanocyte, CD8+ T cell

## Abstract

Vitiligo, an autoimmune skin disorder characterized by melanocyte loss, has long been associated with sympathetic nervous system activity. Clinical observations have suggested links between psychological stress, sympathetic activation, and vitiligo progression. However, direct experimental evidence for the role of sympathetic nerves in vitiligo development has been lacking. Herein, we employed 6-hydroxydopamine (6-OHDA) to induce sympathetic nerve depletion in mice before vitiligo induction. Sympathetic nerve ablation was confirmed through immunofluorescent staining of tyrosine hydroxylase. Vitiligo progression was assessed by quantifying epidermal melanocytes and CD8+ T cells using whole-mount immunofluorescence staining. The loss of melanocytes and infiltration of CD8+ T cells in vitiligo lesions were comparable between sympathectomized and control mice. Overall, our study suggested that previously observed associations between sympathetic nervous system activity and vitiligo may be concomitant effects rather than causative factors, challenging long-held clinical hypotheses.

## Introduction

Vitiligo is one of the most prevalent autoimmune skin disorders, affecting 0.5 to 2% of the global population (1). It is characterized by the chronic loss of skin pigmentation in a patchy manner, primarily mediated by autoreactive CD8+ T cells (1, 2). Despite extensive research, the precise mechanisms underlying the initiation and progression of vitiligo remain elusive, with various environmental and physiological factors proposed as potential triggers.

The potential role of the nervous system in vitiligo pathogenesis has been a subject of interest for over six decades, dating back to Lerner’s seminal proposal in 1959 (3). Since then, numerous studies have explored the complex relationship between psychological stress, the sympathetic nervous system, and vitiligo.

Psychological stressors have been consistently linked to both the onset and exacerbation of vitiligo. Specific life events, such as the death of a family member or work-related problems, have been identified as potential precipitating factors (4). This connection is particularly intriguing given that mental stress has been shown to significantly increase sympathetic nerve activity.

Early clinical observations provided initial insights into the relationship between sympathetic nerve function and vitiligo. Neurotransmitters and neuropeptides associated with the sympathetic nervous system, including catecholamines, substance P, norepinephrine, and neuropeptide Y, have been found to be elevated in the skin or plasma of vitiligo patients (5–7). These molecules have been implicated in various inflammatory processes or melanocytes dysfunction. Studies have shown that catecholamines and substance P can promote inflammatory conditions in multiple immune cells (8, 9), while norepinephrine and neuropeptide Y increase has been linked to hair depigmentation (10, 11).

Interestingly, the role of the sympathetic nervous system in vitiligo may not be straightforward. While sympathetic activation appears to play a role in vitiligo development, studies have also shown that sympathetic nervous system depletion can increase inflammatory responses in some contexts. For instance, sympathetic depletion has been observed to induce more severe colitis and central nervous system autoimmune disease in mice, mediated by elevated levels of inflammatory markers such as IFN-*γ*, IL-1β, and IL-2 (12, 13).

Despite these various associations and observations, direct evidence demonstrating the impact of sympathetic nerves on vitiligo remains absent. To address this gap, we employed selective sympathetic denervation in a well-characterized vitiligo mouse model.

Our approach utilizes a melanoma-Treg-induced vitiligo mouse model that could activate endogenous autoreactive CD8+ T cells (14). This model is established based on two clinical observations: dysfunction of regulatory T cells (Treg) is commonly observed in vitiligo patients, and some melanoma patients receiving immunotherapy spontaneously develop vitiligo as a side effect (15–17). The model recapitulates key pathological features of human vitiligo, including skin depigmentation, loss of epidermal melanocytes, and skin infiltration of auto-reactive CD8+ T cells (14, 18). While this model may not capture all vitiligo subtypes, particularly those driven by distinct genetic or environmental factors, it provides a robust platform for investigating disease mechanisms and has been extensively validated for mechanistic studies and therapeutic development (18–20).

To achieve sympathetic denervation, we utilized 6-hydroxydopamine (6-OHDA), a selective neurotoxin widely used for sympathetic nerve ablation (21). 6-OHDA preferentially enters sympathetic neurons through dopamine and norepinephrine transporters, where it generates toxic metabolites that induce oxidative stress and subsequent nerve degeneration (22). Due to the specific expression of these transporters, 6-OHDA selectively ablates sympathetic neurons while sparing other cell types with no significant (23). This selectivity has been validated by numerous studies, confirming the reliability and safety of 6-OHDA for sympathectomy (10, 24). Through this approach, we aim to investigate the role of sympathetic nerves in vitiligo progression, potentially providing crucial insights into the complex interplay between the nervous system, immune responses, and pigmentation in vitiligo.

## Materials and methods

### Mice and cell lines

C57BL/6 N mice were purchased from Charles River. Mice were maintained in the National Institute of Biological Sciences, Beijing (NIBS) specific-pathogen-free (SPF) facility in accordance with the Guide for the Care and Use of Laboratory Animals of NIBS. Procedures were approved by the Laboratory Animal Management Committee of NIBS (NIBS2024M011) and were in compliance with all relevant ethical regulations.

B16F10 melanoma cells (ATCC, CRL-6475) were maintained in DMEM medium supplemented with 10% (v/v) fetal bovine serum and 1% (v/v) penicillin–streptomycin at 37°C and 5% CO_2_.

### Experimental design

**Figure 1:**
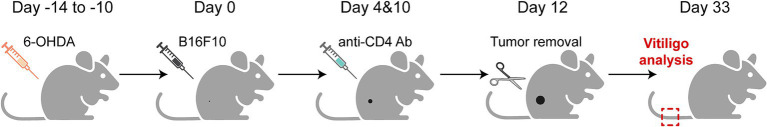
Schematic of the experimental design for sympathetic nerve ablation and vitiligo induction in mice.

This study utilized female mice aged 7–9 weeks. The mice were randomized into control or chemical sympathectomy groups. Figure 1 illustrates the schematic of the experimental design for sympathetic nerve ablation and vitiligo induction in mice.

Sympathetic nerve ablation was performed using freshly prepared 6-OHDA, while control mice received an equivalent volume of the vehicle solution. To investigate vitiligo induction, we utilized a melanoma-Treg-induced vitiligo mouse model as described in previous studies (14, 18).

On day 0, the mice were intradermally inoculated with 2 × 10^5^ B16F10 melanoma cells in the right flank. Then on days 4 and 10, the mice received intraperitoneal injections of CD4 depletion antibody (Bio X Cell, BE0003-1) at a dosage of 10 mg/kg to ablate CD4+ regulatory T cells. By day 12, the developed tumors were surgically removed, and the wounds were closed using suture clips. To evaluate the efficiency of vitiligo induction, whole-mount immunofluorescent staining of epidermal melanocytes and CD8+ T cells was performed on mouse tail skin on day 33 post-induction.

### 6-OHDA preparation and administration

A fresh solution of 6-OHDA (Sigma, H4381) was prepared by dissolving it in 0.9% sterile NaCl containing 0.1% L-ascorbic acid. Mice in the chemical sympathectomy group received intraperitoneal injections of 6-OHDA at a dosage of 100 mg/kg for five consecutive days. Control mice were administered an equivalent volume of the vehicle solution (0.9% sterile NaCl with 0.1% L-ascorbic acid) via the same route. During the treatment period, mice were monitored daily for potential side effects, including changes in body weight, food intake, and general behavior. No significant adverse effects were observed. To confirm the effectiveness of the sympathetic denervation, we conducted tyrosine hydroxylase (TH) immunostaining of skin sections ten days after the final 6-OHDA injection.

### Whole-mount immunofluorescent staining

Tail epidermis was separated from tail skin by incubating in 20 mM EDTA solution and shaking at 80 rpm in 37°C for 1 h. The separated tail epidermis was subsequently fixed with 4% paraformaldehyde in PBS for 10 min, washed three times in PBS for 10 min, permeabilized in 0.3% H_2_O_2_ at −20°C for 20 min, washed three times in PBS for 10 min, blocked with blocking buffer (2% normal donkey serum, 1% BSA, 0.3% Triton in PBS) for 1 h. Then the tail epidermis was incubated at 4°C overnight with 1 μg/mL DAPI and anti-mouse DCT (lab made, 1:3000) and anti-mouse CD8a (eBioscience, cat: 17-0081-83, 1:300) in blocking buffer. After washed with three times in PBS for 15 min, the tail epidermis was incubated for 1 h with secondary antibodies including Donkey Anti-Rabbit Alexa Fluro 488 (1:1000) and Donkey Anti-Rat Alexa Fluor 647 (1:1000) in blocking buffer. The tail epidermis was washed with three times in PBS for 15 min and mounted with 50% glycerol and applied with coverslip.

### Sympathetic nerve immunofluorescent staining

For sympathetic nerve immunofluorescent staining, skin tissues were embedded in frozen OCT compound, cryosectioned to 50 μm thickness, and then fixed for 10 min in 4% paraformaldehyde in PBS. Sections were washed three times in PBS for 10 min, blocked with blocking buffer (2% normal donkey serum, 1% BSA, 0.3% Triton in PBS) for 1 h. Sections were incubated at 4°C overnight with anti-tyrosine hydroxylase antibody (Millipore, cat: AB152, 1:300) and anti-mouse KRT14 (lab made; 1:1000) in blocking buffer. After washed with three times in PBS for 15 min, the sections were incubated for 1 h with secondary antibodies including Donkey Anti-Rabbit Alexa Fluro 488 (1:1000) and Donkey Anti-Rat Alexa Fluor 647 (1:1000) in blocking buffer. The sections were washed with three times in PBS for 15 min and mounted with 50% glycerol-DAPI and applied with coverslip.

### Imaging and image analysis

Fluorescent samples were imaged using a Nikon A1-R, Nikon A1 SIM, or Nikon AX confocal microscope equipped with 10x or 20x objective lenses.

TH+ nerve fiber analysis was performed using ImageJ. Raw image files (.nd2) were loaded into ImageJ, and the Straight Line tool was used to draw a line along the skin or nerve fiber. Measurements were obtained by selecting Analyze > Measure, with results displayed in the Results window.

Melanocyte and CD8+ T cell quantification was analyzed with Imaris software, following previously reported methods (14). Image processing and analysis were performed using Imaris software (Bitplane). After loading raw files into Imaris, fluorescent signals were converted to digital spots using the “Spot” function, with cell diameter parameters set to 15 μm for melanocytes and 7 μm for CD8+ T cells. The Statistics function was used to obtain the total spot count. Sample width and height were measured to calculate area. Cell density was determined by dividing total spots by area.

For spatial analysis of melanocytes and CD8+ T cells, spot position coordinates were exported from Imaris and processed for analysis in RStudio. Scatter plots were generated using the ggplot2 package, and density distributions were visualized using the smoothscatter function.

## Results

### 6-OHDA significantly depletes sympathetic nerves in mice

**Figure 2:**
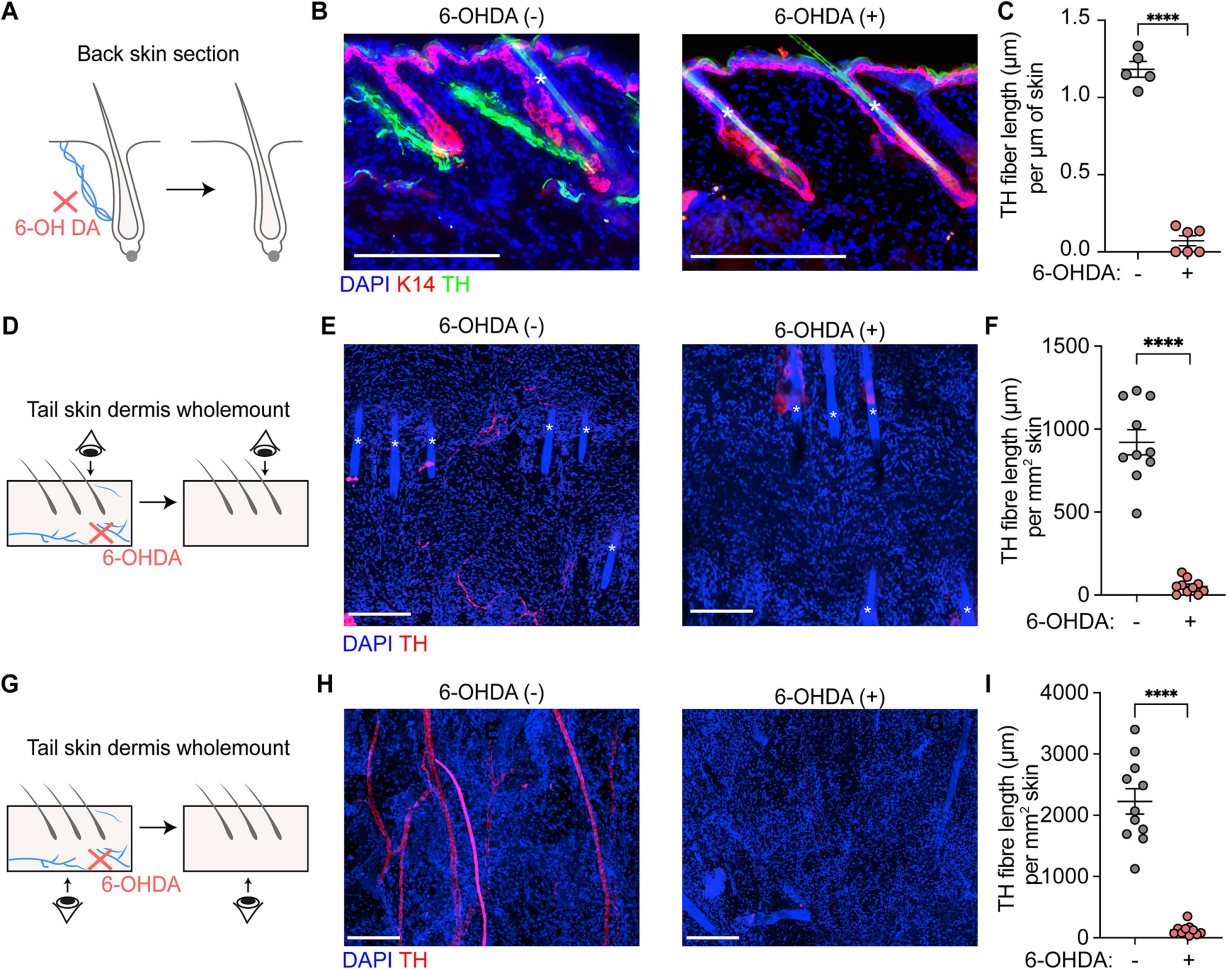
Sympathetic nerve depletion following 6-OHDA treatment. **(A,B)** Scheme and representative section immunofluorescence images of sympathetic nerves in back skin of control and 6-OHDA-treated mice. Tyrosine hydroxylase (TH) serves as a marker of sympathetic nerve. K14 indicates the epidermal cells. The asterisks indicate auto-fluorescent hair shafts. Scale bars = 200 μm. **(C)** Quantification of TH+ sympathetic nerve fiber length per μm skin in control and 6-OHDA-treated mice in back skin. For TH+ sympathetic nerve fiber length per μm skin in control and 6-OHDA-treated mice in back skin: 1.182 ± 0.05 μm/μm skin; 0.07 ± 0.03 μm/μm skin. Data are presented as mean ± SEM. *n* = 5 or 6 mice per group. **** *p* < 0.0001 (unpaired *t*-test). **(D,E)** Scheme and representative wholemount staining images showing the sympathetic nerves distribution in tail upper dermis of control and 6-OHDA-treated mice. The asterisks indicate auto-fluorescent hair shafts. Scale bars = 200 μm. **(F)** Quantification of TH+ sympathetic nerve fiber length per mm^2^ skin in control and 6-OHDA-treated mice in upper dermis of tail skin. For TH+ sympathetic nerve fiber length per mm^2^ skin in control and 6-OHDA-treated mice: 920.50 ± 75.97 μm/mm^2^; 50.01 ± 14.02 μm/mm^2^. Data are presented as mean ± SEM. *n* = 10 random areas from 2 mice for each condition. **** *p* < 0.0001 (unpaired *t*-test). **(G,H)** Scheme and representative wholemount staining images showing the sympathetic nerves distribution in tail lower dermis of control and 6-OHDA-treated mice. Scale bars = 200 μm. **(I)** Quantification of TH+ sympathetic nerve fiber length per mm^2^ skin in control and 6-OHDA-treated mice in lower dermis of tail skin. For TH+ sympathetic nerve fiber length per mm^2^ skin in control and 6-OHDA-treated mice: 2226 ± 207.10 μm/mm^2^; 126.6 ± 28.65 μm/mm^2^. Data are presented as mean ± SEM. *n* = 10 random areas from 2 mice for each condition. **** *p* < 0.0001 (unpaired *t*-test).

Tyrosine hydroxylase (TH) serves as a specific marker for sympathetic nerve fibers. Fluorescent immunostaining with anti-TH antibody in the skin (Figure 2A) revealed the distribution of sympathetic nerves. These nerves were found to be located between the epidermis and hair follicles, forming tight connections with the bulge region. In the dermis, TH+ sympathetic nerve fibers converged into single nerve fibers.

Tyrosine hydroxylase (TH) serves as a specific marker for sympathetic nerve fibers. Fluorescent immunostaining with anti-TH antibody in the skin revealed the distribution of sympathetic nerves. In back skin, these nerves were found to be located between the epidermis and hair follicles, forming tight connections with the bulge region (Figure 2B). In the tail skin, TH+ sympathetic nerve fibers were present in both the upper and lower dermis (Figures 2E,H). In the upper dermis, sympathetic nerve fiber display terminal-like characteristics, indicated by their fine and short structure. In lower dermis, the sympathetic nerve fibers are thicker and longer, with some fibers appears branching.

Daily administration of 6-OHDA for five consecutive days resulted in marked depletion of TH+ sympathetic nerve fibers in both back and tail skin. In back skin, TH+ sympathetic nerve fibers were nearly undetectable following 6-OHDA treatment (Figures 2A,B), with fiber lengths measuring 1.18 ± 0.05 μm per μm of skin in control mice compared to 0.07 ± 0.03 μm per μm of skin in treated mice. In the lower dermis of tail skin, treatment with 6-OHDA caused a reduction in fiber length from 2,226 ± 207.10 μm per mm^2^ of skin to 126.6 ± 28.65 μm per mm^2^ of skin (Figures 2D–F). A similar decline in TH+ sympathetic nerve fiber length was evident in the upper dermal region of the tail skin; control mice exhibited lengths of 920.50 ± 75.97 μm/mm^2^, while treated mice showed a significant reduction to 50.01 ± 14.02 μm/mm^2^ (Figures 2G–I).

### Sympathetic nerve depletion alone does not alter epidermal melanocytes or CD8+ T cells

**Figure 3:**
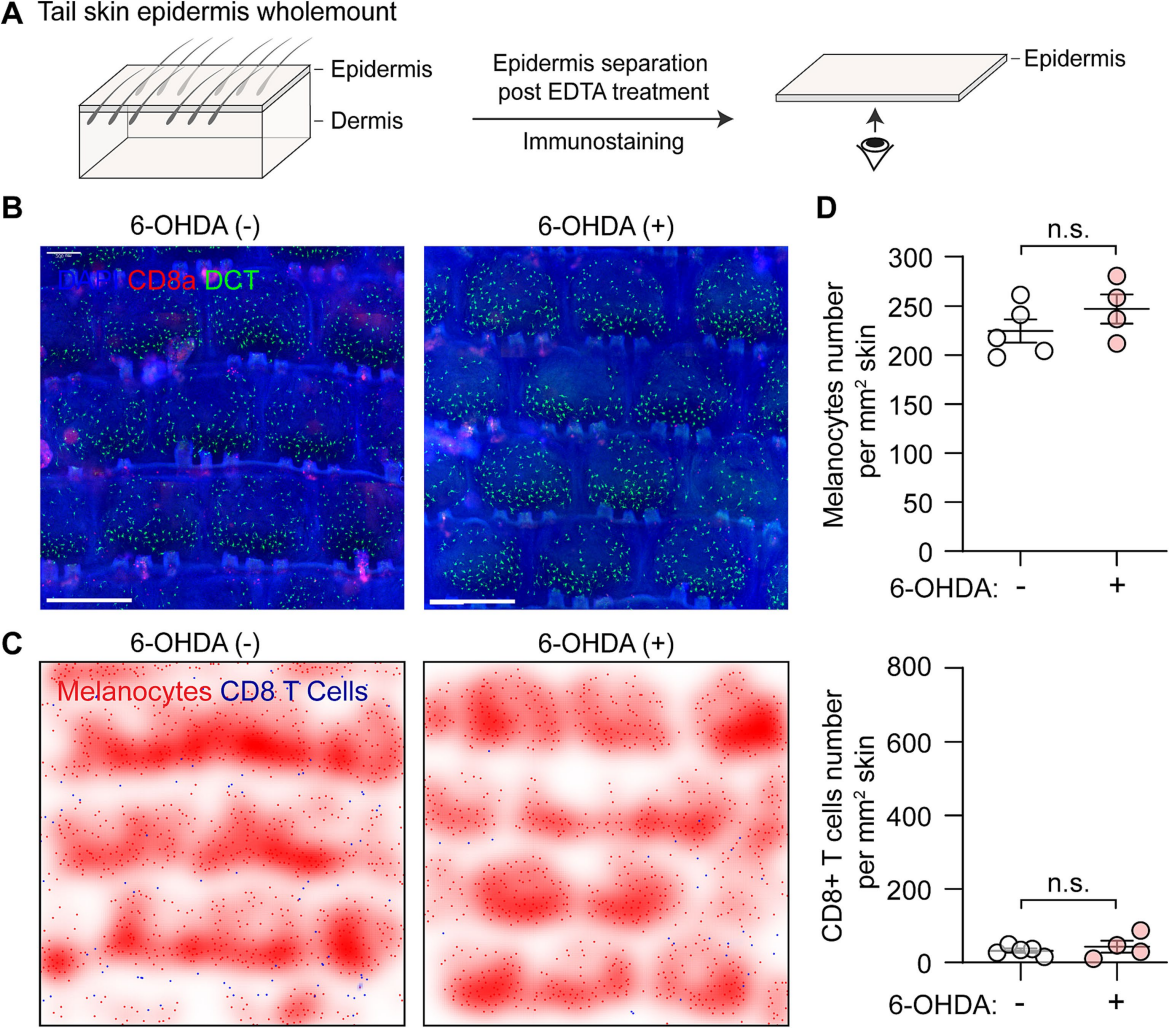
Distribution of melanocytes and CD8+ T cells following chemical sympathectomy. **(A)** Scheme illustrating wholemount staining procedure for the tail epidermis basal layer, where melanocytes reside. **(B)** Representative whole-mount immunofluorescence staining images of epidermal melanocytes (green) and CD8+ T cells (red) in control and sympathectomized mice. Scale bar = 500 μm. **(C)** Smoothed density plots illustrating the distribution pattern of melanocytes and CD8+ T cells in control and sympathectomized mice. **(D)** Quantification of epidermal melanocytes and CD8+ T cells per unit area (cells/mm^2^) in control and sympathectomized mice. For epidermal melanocytes in control mice and sympathectomized mice: 224.30 ± 11.87 cells/mm^2^; 246.90 ± 14.77 cells/mm^2^. For epidermal CD8+ T cells in control mice and sympathectomized mice: 32.65 ± 5.58 cells/mm^2^; 43.50 ± 16.47 cells/mm^2^. Data are presented as mean ± SEM. *n* = 4 or 5 mice per group. n.s. = not significant (unpaired *t*-test).

To evaluate the potential effect of sympathetic nerve ablation on epidermal melanocytes and CD8+ T cells, we performed whole-mount fluorescent immunostaining of tail epidermis. The epidermis was carefully separated following EDTA treatment, and immunostained images were captured from the dermal side. This approach allows for clear visualization of the basal layer of the epidermis, where melanocytes are located (Figure 3A). Dopachrome tautomerase (DCT) was used as a typical melanocyte marker, while CD8a was used to label CD8+ T cells (Figure 3B). To clearly demonstrate the distribution pattern of CD8+ T cells and melanocytes, we further processed the immunostaining images by converting the signals to digital spots and creating density plots (Figure 3C).

In control mice, melanocytes were evenly distributed in the epidermis at a density of approximately 225/mm^2^ of skin, and CD8+ T cells were rarely detected at a low density of 32.65 ± 5.58 cells/mm^2^ (Figure 3D). After chemical sympathectomy with 6-OHDA, the immunostaining data revealed that the numbers of melanocytes (246.90 ± 14.77 cells/mm^2^) and CD8+ T cells (43.50 ± 16.47 cells/mm^2^) were comparable to those in control mice (Figure 3C).

### Sympathetic nerve depletion does not affect the progression of vitiligo

Whole-mount immunofluorescence staining provides an effective method for monitoring vitiligo development. By acquiring large-scale images, we can characterize vitiligo progression based on quantification of the total cell numbers for melanocytes and CD8+ T cells.

**Figure 4:**
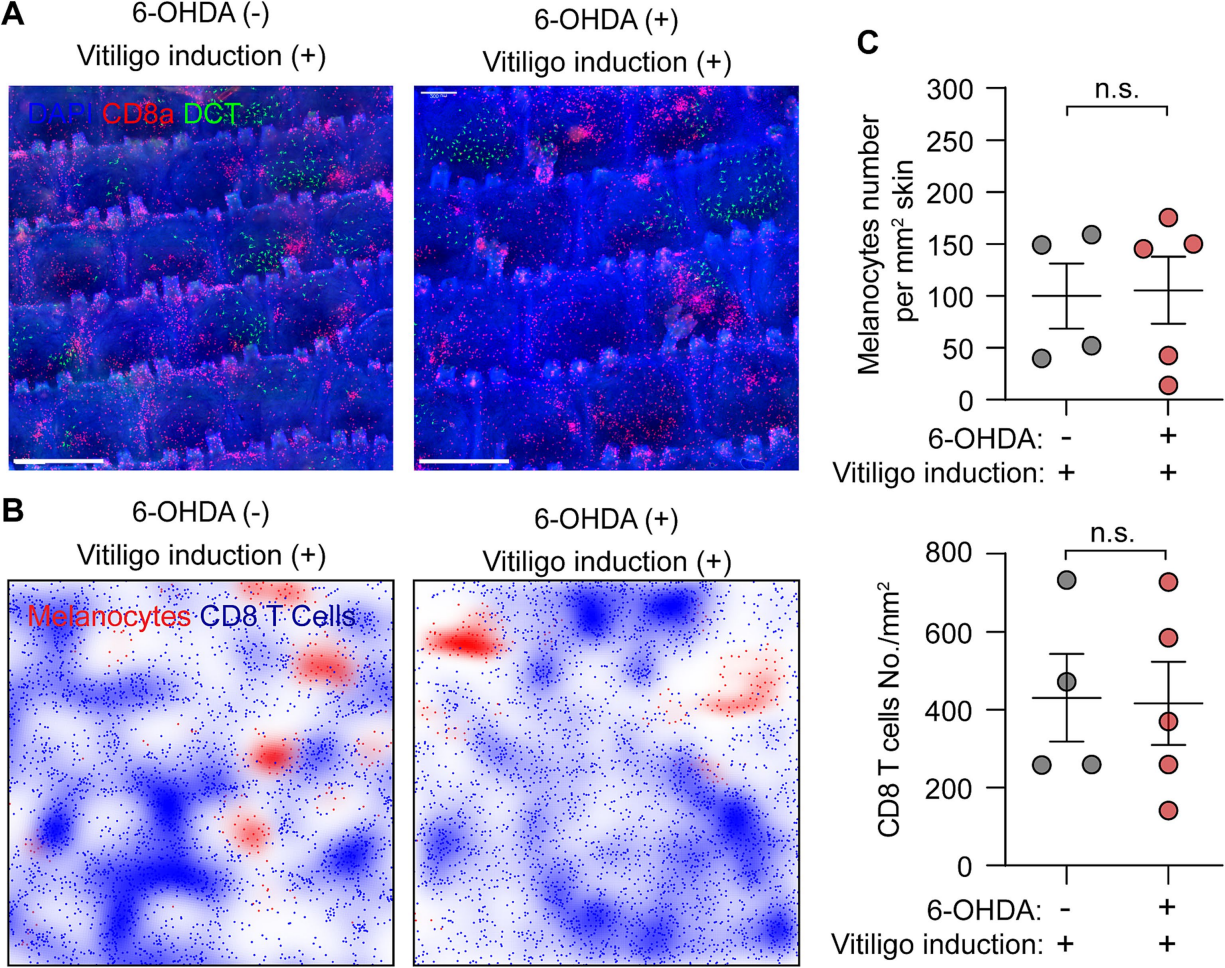
Assessment of vitiligo characteristics following sympathetic nerve depletion. **(A,B)** Representative **(A)** whole-mount immunofluorescence staining images and **(B)** density plots of epidermal melanocytes (green) and CD8+ T cells (red) in control and sympathectomized mice 33 days post-vitiligo induction. Scale bar = 500 μm. **(C)** Quantification of epidermal melanocytes and CD8+ T cells in control and sympathectomized mice 33 days post-vitiligo induction. For epidermal melanocytes in control mice and sympathectomized mice 33 days post-vitiligo induction: 99.89 ± 31.36 cells/mm^2^; 105.40 ± 32.18 cells/mm^2^. For epidermal CD8+ T cells in control mice and sympathectomized mice 33 days post-vitiligo induction: 430.7 ± 112.60 cells/mm^2^; 416.50 ± 106.80 cells/mm^2^. Data are presented as mean ± SEM. *n* = 4 or 5 mice per group. n.s. = not significant (unpaired *t*-test).

At Day 33 post-vitiligo induction, control mice exhibited significant loss of melanocytes and abundant infiltration and aggregation of CD8+ T cells in the epidermis of the tail skin, as evidenced by the immunostaining images and density plots (Figures 4A,B). These observations are consistent with the histological features of vitiligo lesional skin (1, 2). Quantitative analysis revealed comparable changes between control and sympathectomized groups: melanocyte density decreased markedly to 99.89 ± 31.36 cells/mm^2^ in controls and 105.40 ± 32.18 cells/mm^2^ in sympathectomized mice, while CD8+ T cell density rose significantly to 430.7 ± 112.60 cells/mm^2^ in controls and 416.50 ± 106.80 cells/mm^2^ in sympathectomized mice (Figure 4C). No significant differences were observed between the groups.

Notably, the ablation of sympathetic nerves did not affect the loss of melanocytes or the infiltration and aggregation of CD8+ T cells in vitiligo-induced mice (Figures 4A–C). These results suggest that sympathetic nerve depletion does not influence the progression of vitiligo in mice.

## Discussion

Our study aimed to investigate the role of sympathetic nerves in vitiligo progression using a melanoma-Treg-induced mouse model. This model activates endogenous autoreactive CD8+ T cells, mimicking the autoimmune nature of vitiligo. By employing 6-OHDA to induce chemical sympathectomy, we sought to directly examine the impact of sympathetic nerve depletion on vitiligo development. Contrary to expectations based on previous literature, our results demonstrate that sympathetic nerve ablation did not significantly alter the course of vitiligo progression in this mouse model.

For decades, clinical observations have suggested a potential link between sympathetic nervous system activity and vitiligo development or progression. Morrone et al. (25) reported significantly increased urine levels of catecholamines in vitiligo patients compared to controls. Elevated neuropeptide levels, such as neuropeptide Y (NPY) and calcitonin gene-related peptide (CGRP), have been detected in the marginal areas of vitiligo lesions (6). Additionally, keratinocytes cultured from vitiligo-affected skin exhibit increased expression of *β*-adrenergic receptors (26). The clinical association between psychological stress and vitiligo progression has also been documented (27). Recently, Zhang et al. (10) demonstrated in murine models that psychological stress-induced sympathetic nerve activation can lead to melanocyte loss via adrenergic signaling pathways. However, direct evidence supporting involvement of sympathetic nerve in vitiligo pathogenesis remains lacking.

While clinical observations suggest potential sympathetic nervous system involvement in vitiligo progression, several studies challenge this hypothesis. Chanco-Turner and Lerner (28) found no differential sympathetic response between vitiligo lesions and normal skin following neurohormone injection. Additionally, a documented case of sympathectomy failed to restore pigmentation in affected areas (29). A larger histological study examined dermal nerves in 74 vitiligo patients and found nerve degeneration in 78% of cases, suggesting neural impairment rather than hyperactivity as a potential mechanism (30). Furthermore, Wu et al. (31) reported no significant differences in plasma levels of adrenaline and noradrenaline between vitiligo patients and healthy controls. These conflicting findings suggest that the relationship between sympathetic innervation and vitiligo pathogenesis may be more complex than initially proposed.

The lack of effect observed following sympathetic nerve ablation in our mouse model suggests that many clinical observations linking sympathetic nervous system activity to vitiligo may be concomitant effects of the disease process rather than causative factors. This interpretation challenges prevailing theories and necessitates a re-evaluation of the role of sympathetic nerves in vitiligo pathogenesis.

Furthermore, our findings have implications for understanding other vitiligo phenomena previously thought to be related to sympathetic nerve activity. For instance, the pathogenesis of segmental vitiligo, often hypothesized to involve neurogenic factors, may need to be reconsidered. Our results suggest that other mechanisms, independent of sympathetic nerve activity, may be involved in these specific vitiligo presentations.

It is important to note, however, that significant differences exist between mouse and human physiology. While our study provides valuable insights into the potential role of sympathetic nerves in vitiligo, these results should be interpreted cautiously when considering human vitiligo. The complexity of human skin innervation, immune responses, and melanocyte biology may lead to different outcomes in human patients compared to our mouse model.

Given these considerations, our study underscores the need for further research to elucidate the complex mechanisms underlying vitiligo. Future studies should aim to investigate potential alternative mechanisms for vitiligo phenomena previously attributed to sympathetic nerve activity and explore the role of other components of the nervous system in vitiligo pathogenesis.

In conclusion, our study challenges existing paradigms regarding the role of sympathetic nerves in vitiligo and opens new avenues for investigation. By providing the first experimental evidence that sympathetic nerve ablation does not affect vitiligo progression in mice, we highlight the need for a revised understanding of neuroimmune interactions in this complex disorder. These findings not only contribute to our fundamental understanding of vitiligo pathogenesis but also have potential implications for future therapeutic approaches.

## Data Availability

The raw data supporting the conclusions of this article will be made available by the authors, without undue reservation.
