# Supplementary material for: Local sympathetic nerve depletion does not alter vitiligo progression in a mouse model
Source: Front Med (Lausanne). 2025 Jan 31;12:1466996. doi: 10.3389/fmed.2025.1466996 (PMC11825348; doi:10.3389/fmed.2025.1466996)
Supplement: Supplementary file 1 [file Table_1.DOCX]

Supplementary Table S1: Studies Disconfirm the Role of Sympathetic Nerve Activity in Vitiligo Pathogenesis

| Reference | Experiment | Conclusion |
| --- | --- | --- |
| Lerner, et al., 1966 | A 29-year-old man with vitiligo was treated by sympathectomy. | Sympathectomy treatment did not reverse pigmentation in vitiligo areas. |
| Samuel et al., 1980 | NA | Local hyperpigmentation followed autosympathectomy related to a Pancoast tumor. |
| Turner and Lerner, 1965 | Injection of sympathetic neurohormoene acetylcholine and epinephrine to test the sympathetic response. | Vitiligo lesional skin and normal skin both had a similar sweat production post injection. |
| Gokhale and Mehta, 1983 | Histological study on dermal nerves and nerve endings of vitiligo skin from 74 patients. | Nerve degeneration is found in 58/74 vitiligo patients lesion skin. |
| Wu, et al., 2000 | Cutaneous blood flow test, sympathetic neurohormone detection on segmental vitiligo, non-segmental vitiligo patients, and healthy individuals. | Cutaneous blood flow is higher in segmental vitiligo lesion, but not changed in non-segmental vitiligo skin;  Plasma level of adrenaline and noradrenaline have no significant differences across all three groups. |
